# A new membrane anatomy-oriented classification of radical surgery for rectal cancer

**DOI:** 10.1093/gastro/goad069

**Published:** 2023-12-21

**Authors:** Jiaqi Wang, Hailong Liu, Ajian Li, Huihong Jiang, Yun Pan, Xin Chen, Lu Yin, Moubin Lin

**Affiliations:** Department of General Surgery, Yangpu Hospital, Tongji University School of Medicine, Shanghai, P. R. China; Institute of Gastrointestinal Surgery and Translational Medicine, Tongji University School of Medicine, Shanghai, P. R. China; Department of General Surgery, Yangpu Hospital, Tongji University School of Medicine, Shanghai, P. R. China; Institute of Gastrointestinal Surgery and Translational Medicine, Tongji University School of Medicine, Shanghai, P. R. China; Department of General Surgery, Yangpu Hospital, Tongji University School of Medicine, Shanghai, P. R. China; Department of General Surgery, Yangpu Hospital, Tongji University School of Medicine, Shanghai, P. R. China; Institute of Gastrointestinal Surgery and Translational Medicine, Tongji University School of Medicine, Shanghai, P. R. China; Institute of Gastrointestinal Surgery and Translational Medicine, Tongji University School of Medicine, Shanghai, P. R. China; Institute of Gastrointestinal Surgery and Translational Medicine, Tongji University School of Medicine, Shanghai, P. R. China; Department of General Surgery, Shanghai Tenth People’s Hospital, Tongji University School of Medicine, Shanghai, P. R. China; Department of General Surgery, Yangpu Hospital, Tongji University School of Medicine, Shanghai, P. R. China; Institute of Gastrointestinal Surgery and Translational Medicine, Tongji University School of Medicine, Shanghai, P. R. China

**Keywords:** rectal cancer, surgical classification, radical surgery, membrane anatomy

## Abstract

For patients with different clinical stages of rectal cancer, tailored surgery is urgently needed. Over the past 10 years, our team has conducted numerous anatomical studies and proposed the “four fasciae and three spaces” theory to guide rectal cancer surgery. Enlightened by the anatomical basis of the radical hysterectomy classification system of Querleu and Morrow, we proposed a new classification system of radical surgery for rectal cancer based on membrane anatomy. This system categorizes the surgery into four types (A–D) and incorporates corresponding subtypes based on the preservation of the autonomic nerve. Our surgical classification unifies the pelvic membrane anatomical terminology, validates the feasibility of classifying rectal cancer surgery using the theory of “four fasciae and three spaces,” and lays the theoretical groundwork for the future development of unified and standardized classification of radical pelvic tumor surgery.

## Introduction

As the “golden standard” of rectal cancer surgery, total mesorectal excision (TME) is the fundamental principle that must be adhered to during operation, and the surgical plane between the visceral fascia and the parietal fascia is regarded as the “holy plane” [1–3]. Although there are various techniques and approaches available for rectal cancer surgery, TME remains the only radical surgical option in terms of surgical plane and extent of clearance. This is not in line with the principle of individualized treatment, as TME is the only available radical surgery for rectal cancer, regardless of the tumor size, location, or clinical stage. Therefore, it is necessary to establish a range of surgical procedures beyond Heald’s TME to tailor rectal cancer surgery [4]. In contrast, the Querleu–Morrow (Q–M) classification [5] defines four types of radical hysterectomies for cervical cancer, with each type tailored to the tumor stage, resulting in individualized surgery. Given that the Q–M classification of cervical cancer is established based on anatomy [5], we propose a new classification system of radical surgery for rectal cancer from the perspective of membrane anatomy. The so-called membrane anatomy mainly studies various membrane structures of the human body related to surgery, such as the peritoneum, fasciae, mesenteries, and ligaments. The concept of membrane anatomy originated from Heald’s TME procedure and is currently prevailing in China [6, 7]. Nowadays, membrane anatomy–guided surgery has been widely used in clinics to treat various tumors and thus has different names, such as fascial anatomy [8], mesenteric anatomy [9], and space anatomy [10].

It should be emphasized that the new classification put forth in this paper is solely grounded in anatomy, which will undoubtedly stir up controversy. However, as David Cibula pointed out, only if one classification system is established and each type of procedure is precisely standardized can the oncological outcome or morbidity be evaluated to judge the accuracy of the classification [11]. The most popular Q–M classification of radical hysterectomy was also originally established based on anatomy [5] and has been included in the National Comprehensive Cancer Network (NCCN) clinical practice guidelines for cervical cancer [12].

## Membrane anatomical basis for classification of radical rectal cancer surgery

Pelvic anatomical terminologies need to be unified and defined in the context of membrane anatomy to elucidate the foundational anatomical principles underlying the classification of radical hysterectomy and subsequently apply them to the classification of radical rectal cancer surgery. Moreover, only through the establishment of well-approved membrane anatomy using the unified anatomical terminology can we elucidate the anatomical basis of different rectal cancer surgical procedures at present, such as TME with preservation of urogenital fascia [13], TME with preservation of Denonvillier’s fascia [14], fascia-oriented lateral lymph node dissection [15], “fascial space priority” lateral lymph node dissection [16], “two spaces” lateral lymph node dissection [17], and three spaces dissection for rectal cancer [18].

The pelvic membrane anatomy is actually a 3D structure including the fasciae, spaces, blood vessels, and nerves. We summarized the anatomy of the pelvic membrane as the theory of “four fasciae and three spaces” [6, 13, 17]. In brief, three spaces are formed laterally to the rectum by the fascia propria of the rectum, the deep layer of the urogenital fascia, the superficial layer of the urogenital fascia, and the parietal fascia, while the blood vessels and nerves are located in the fasciae and spaces.

### Fascia propria of the rectum

The fascia propria of the rectum presents as a translucent layer of the thin fascia surrounding the rectum, its vessels, and lymphatic and adipose tissues, which is a separate layer from the visceral fascia [17].

### Urogenital fascia

The urogenital fascia is the same fascia as the “visceral fascia” described by Takahashi *et al*. [18] and Heald *et al*. [4] and is the same anatomical structure of various anatomical terminologies appearing in the literature, such as the mesoureter, uretero-hypogastric fascia, prehypogastric nerve fascia, hypogastric nerve sheath, urogenital-hypogastric sheath, uterosacral ligament, and vesicouterine ligament [19–21].

The urogenital fascia is a two-layered structure with deep and superficial layers that contain the hypogastric nerve (HN) and the ureter. The terms “superficial” or “deep” are defined relative to the skin surface. The urogenital fascia lies posterolateral to the rectum, providing a hammock-like support to the rectum.

**Figure 1.**
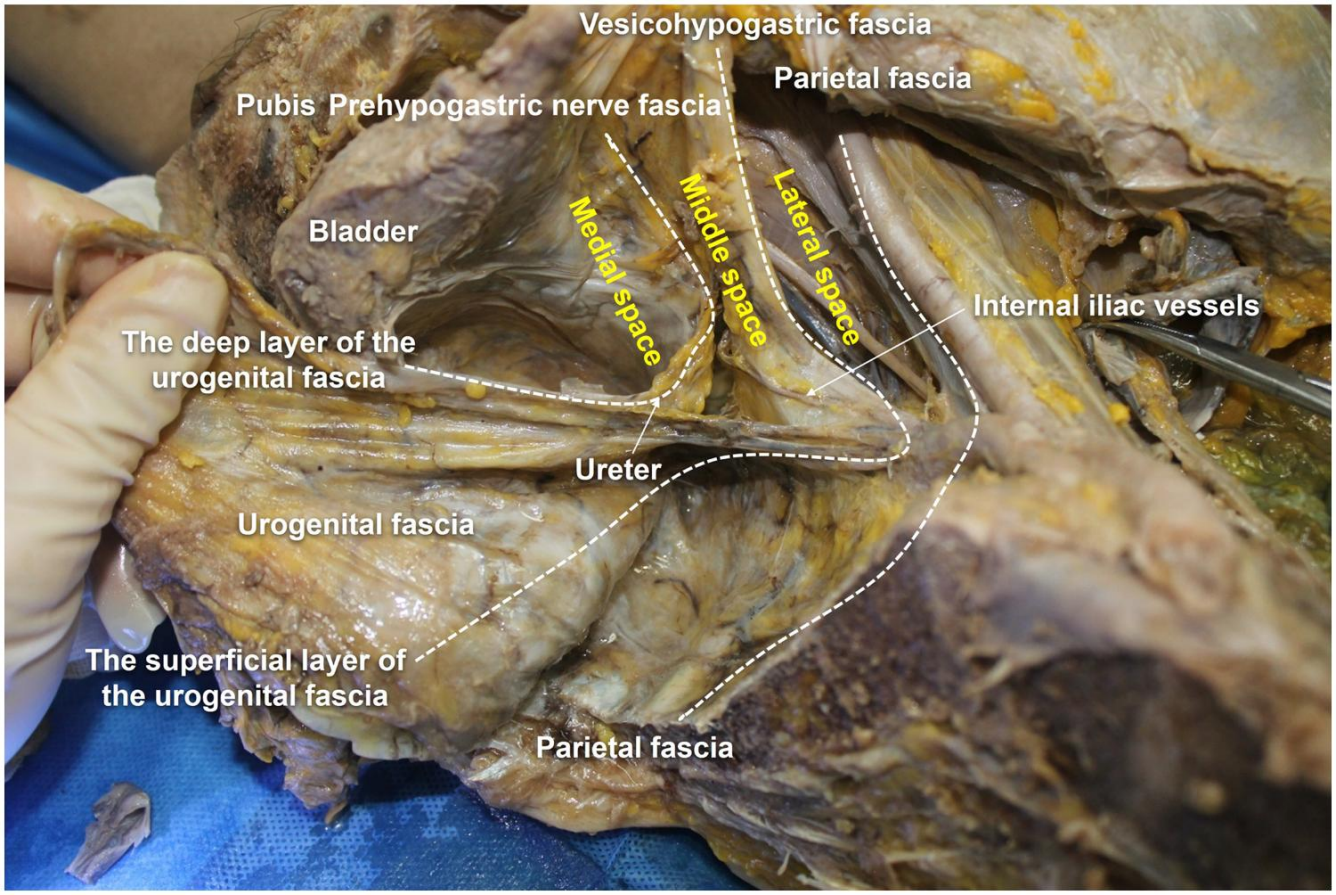
Two-layered structure of the urogenital fascia

The deep layer extends anterolaterally overlying the ventral surface of the HN, and is thus known as the prehypogastric nerve fascia; the superficial layer extends anterolaterally and covers the dorsal surface of visceral branches of the internal iliac artery, which is also called the vesico-hypogastric fascia (Figure 1). The HN and the umbilical artery (the first branch of the internal iliac artery) constitute the anatomical landmarks of the deep and superficial layers of the urogenital fascia, respectively.

### Parietal fascia

The parietal fascia includes four parts: the obturator fascia, the piriformis fascia, the superior fascia of the pelvic diaphragm, and the presacral fascia.

### Fascial spaces

Three spaces are formed by four layers of fasciae around the rectum. The medial space is located between the fascia propria of the rectum and the deep layer of the urogenital fascia; the middle space is located between the deep and superficial layers of the urogenital fascia; and the lateral space is located between the superficial layer of the urogenital fascia and the parietal fascia [17, 21].

**Figure 2.**
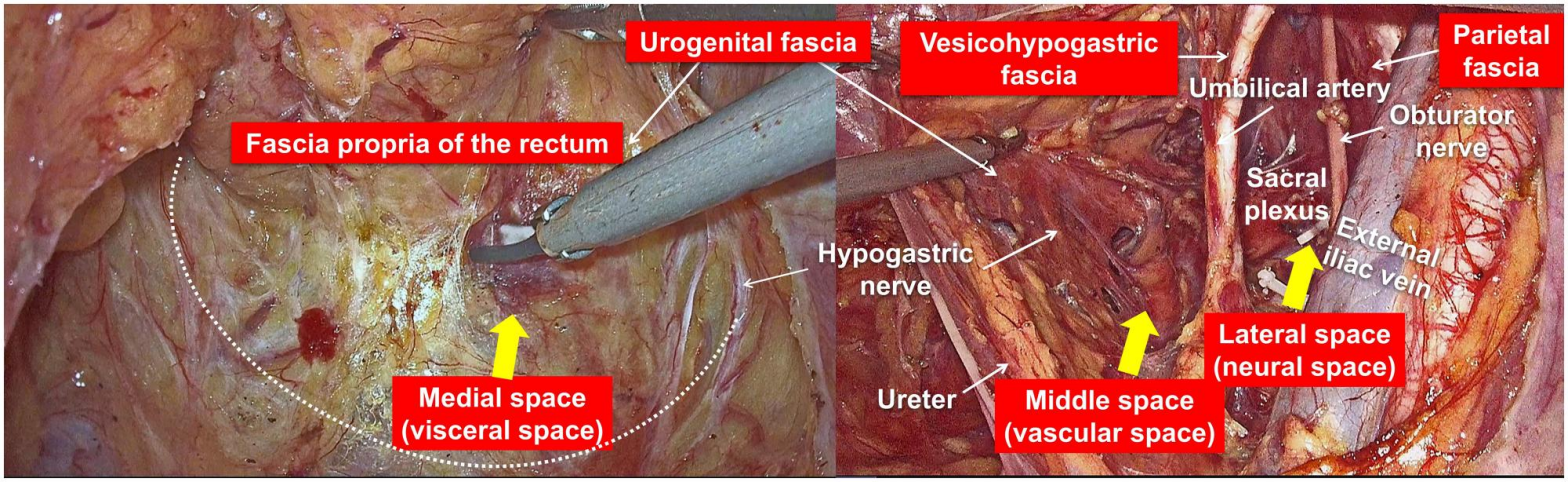
Distribution and composition of three fascial spaces

Since the medial space lacks essential vessels and nerves, it is also referred to as the “visceral space” (Figure 2). The middle space mainly contains vascular structures; almost all the visceral branches of the internal iliac vessels are located in this space, such as the uterine artery, middle rectal artery, and superficial and deep uterine veins. As a result, it is referred to as a “vascular space.” The middle space also contains the inferior hypogastric plexus (IHP) and its efferent branches, the HN and pelvic splanchnic nerves (PSNs), with the IHP and the HN lying close to the deep layer of the urogenital fascia (Figure 2). The lateral space is mainly composed of neural structures, such as the lumbosacral trunk, sacral nerves, sacral plexus, obturator nerves, and sciatic nerves, and can be referred to as the “neural space” (Figure 2).

### Distribution of the pelvic autonomic nerve

**Figure 3.**
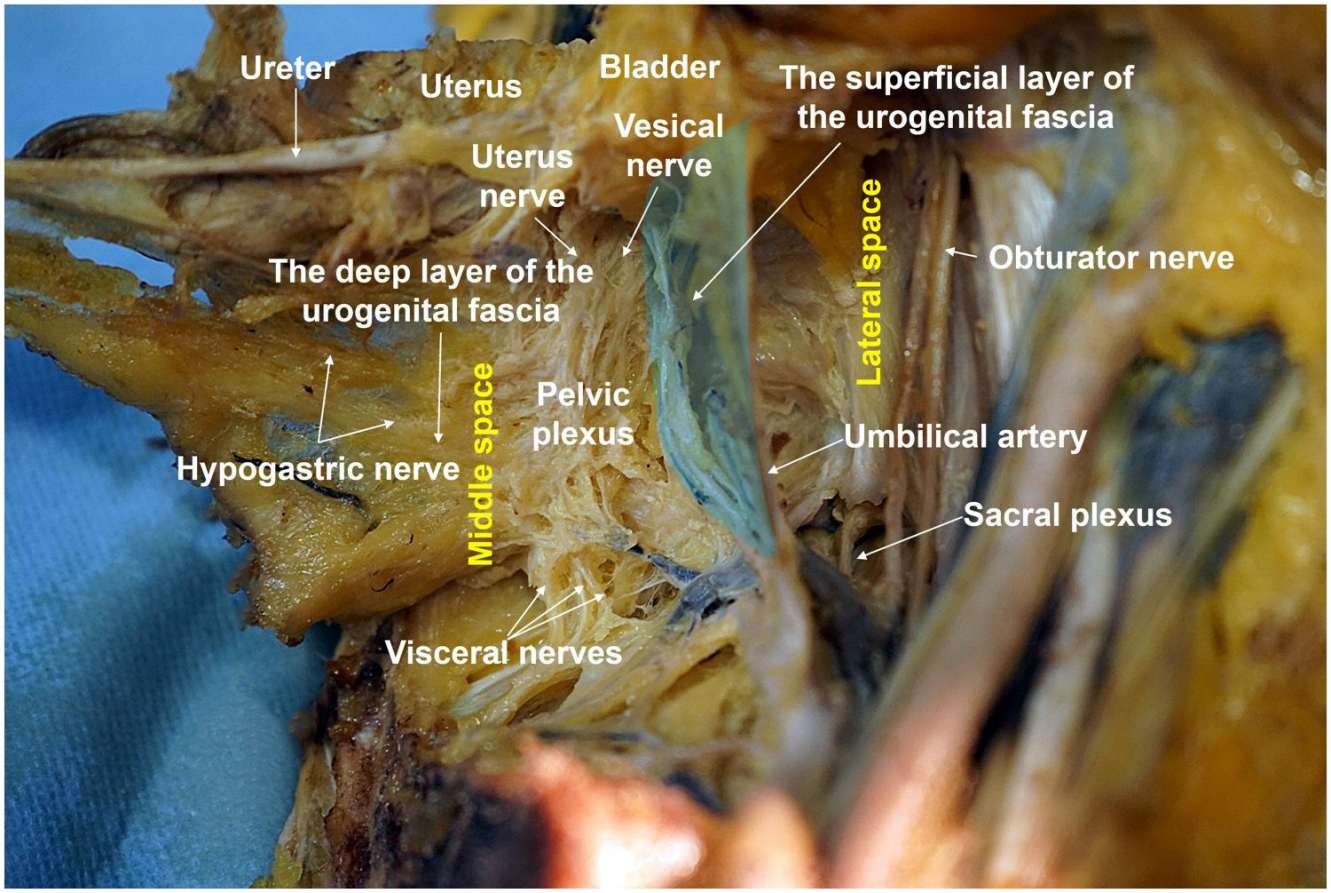
Relationship between the urogenital fascia and nerves

The HN runs between two layers of the urogenital fascia and is separated into deep and superficial layers at the level of the lateral side of the ureter. The deep layer fuses anteriorly with Denonvilliers’ fascia. Exactly external to the junction of the deep layer and Denonvilliers’ fascia, the PSNs join the HN to form the IHP [20]. The IHP gives off the rectal nerve branches via the medial space, the uterine nerve branches (UNB), and the vesical nerve branches (VNB) along the deep layer of the urogenital fascia (Figure 3).

## The rationality to classify radical rectal cancer surgery by membrane anatomy

**Figure 4.**
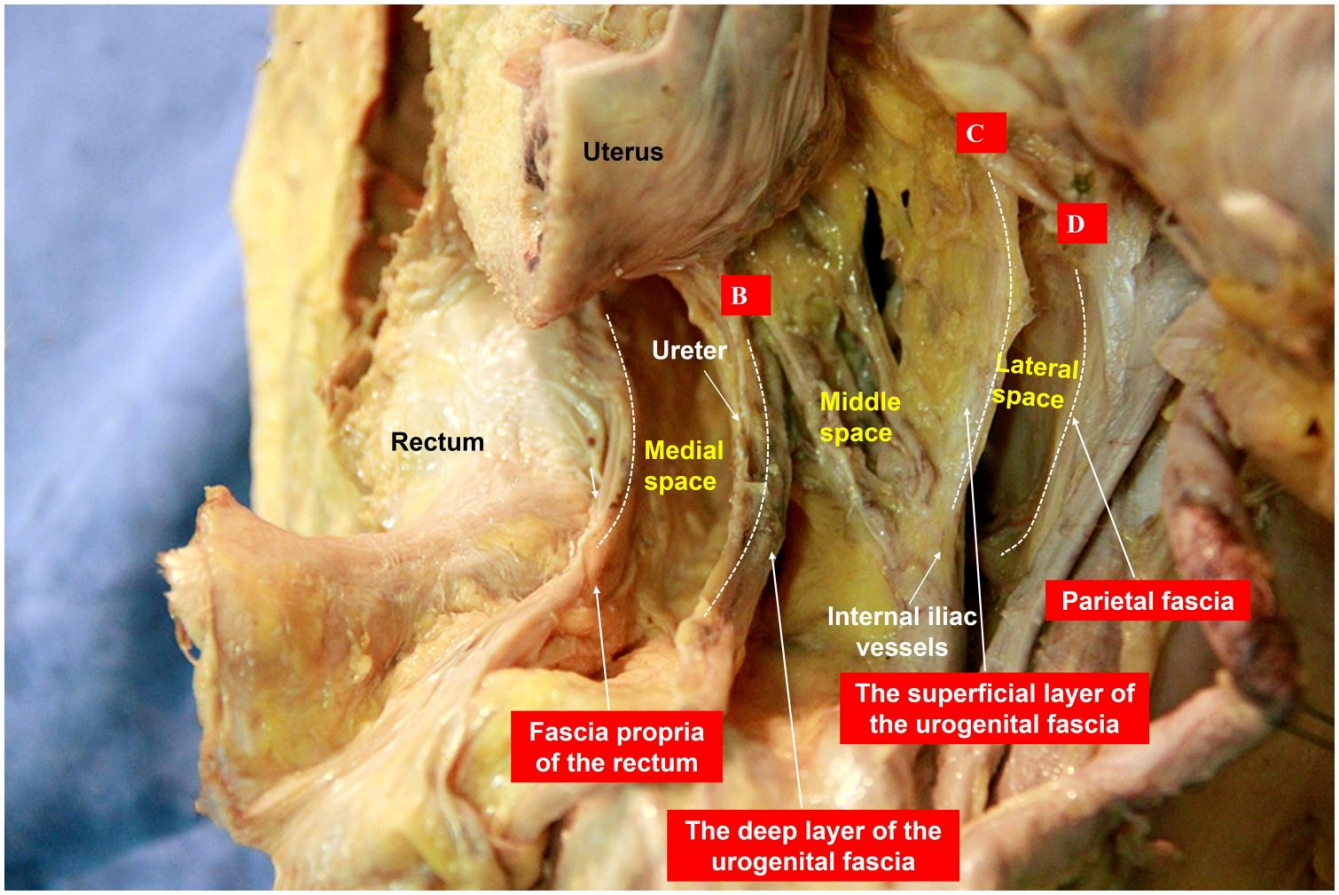
Different range of mesenteries identified from anatomical morphology (white dashed line). B, C, and D represent surgery types B, C, and D of the Q–M classification, respectively.

In 2008, Querleu and Morrow established the classification of radical hysterectomy based solely on the lateral extent of resection, i.e. Q–M classification [5]. At that time, there was no concept of membrane anatomy. Therefore, Q–M classification uses stable anatomical landmarks, such as the organs or vessels, as the key and sole parameter for distinguishing between types of radical hysterectomy. For example, the lateral resection extent of surgery types A–D is between the cervix and the ureter, at the level of the ureter, medial to the internal iliac vessels and the pelvic wall, respectively (Figure 4). The subjectivity in defining resection margins between types of radical hysterectomy may raise questions about the rationality of the classification. However, from the standpoint of membrane anatomy, the ureter, internal iliac vessels, and pelvic wall muscles cover the deep layer of the urogenital fascia, the superficial layer of the urogenital fascia, and the parietal fascia, respectively, which are actually three of the “four fasciae” proposed by the author. The clearance extents of surgery types B–D correspond to the “three spaces” of our theory as well (Figure 4). Therefore, the “four fasciae and three spaces” theory is actually the underlying basis for the Q–M classification of cervical cancer. It is also possible to use the theory of membrane anatomy to classify radical surgery for rectal cancer.

The “four fasciae and three spaces” theory is not only a concrete reflection of Sato’s “onion-like structure” theory of trunk fascia [22], but also an extension of the “mesenteric envelope” theory [23, 24]. The theory behind TME is that all lymphovascular cancer spread will initially, at least, remain within the embryologic hindgut “envelope” because the mesorectal fascia constitutes an almost impenetrable barrier to carcinoma spread [25]. In terms of the “four fasciae and three spaces” theory, the area surrounded by each fascia can be imagined as a “mesentery” and the “four fasciae” thus form “mesenteries” of different sizes and scope, while the “three spaces” constitute the “holy plane” of the corresponding “mesentery” excision.

## Proposed classification of radical rectal cancer surgery based on membrane anatomy

Based on the theory of “four fasciae and three spaces,” the author describes four types of radical surgery for rectal cancer (A–D), adding a few subtypes that consider nerve preservation when necessary (Table 1).

**Table 1.** Surgical classification of radical surgery for rectal cancer based on membrane anatomy

Classification	Surgical approach	Surgical plane
Type A	Total mesorectal excision with urogenital fascia preservation	Medial space
Type B	Classical total mesorectal excision	Middle space
Type B1	—With pelvic autonomic nerve preservation	
Type B2	—Without pelvic autonomic nerve preservation	
Type C	Extended total mesorectal excision	Lateral space
Type C1	—With pelvic autonomic nerve preservation	
Type C2	—Without pelvic autonomic nerve preservation	
Type D	Laterally extended resection	Lateral space
Type D1	—Laterally extended radical resection	
Type D2	—Lateral pelvic exenteration	

### Type A: TME with urogenital fascia preservation

**Figure 5.**
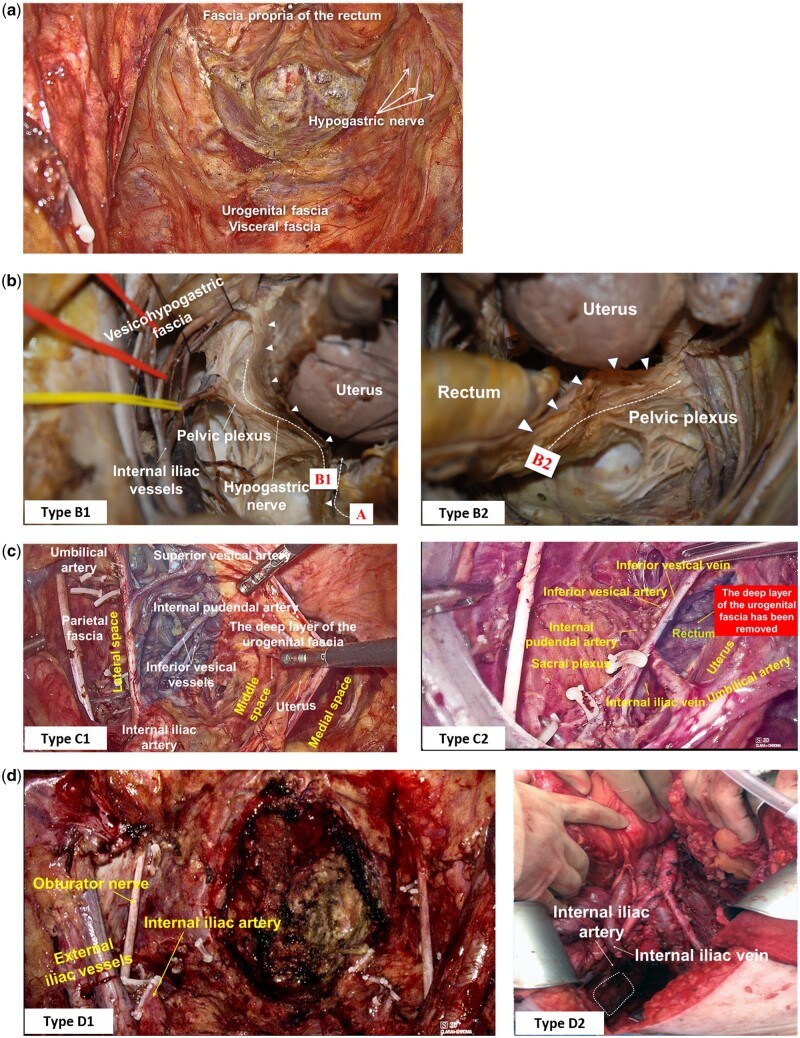
Surgical classification of radical rectal cancer surgery based on membrane anatomy. (A) Type A surgery; (B) type B surgery; (C) type C surgery; (d) type D surgery. The white dashed area indicates a partial excision of the internal obturator muscle.

The surgical plane was located in the medial space and the “fascial envelope” formed by the fascia propria of the rectum was resected (Figure 5a). This tailored procedure is suitable for very early tumors classified by the European Society of Medical Oncology (ESMO) guidelines, including cT1N0 with adverse histopathological factors (sm 2, G3, V1, L1) and early cT1–cT2, cN0 tumors.

### Type B: classical TME

There are two subtypes according to whether or not the autonomic nerve is preserved. Type B1 is the type with pelvic autonomic nerve preservation and type B2 is the type without pelvic autonomic nerve preservation

The ESMO guidelines recommend neoadjuvant treatment for patients with middle-stage lower rectal cancer cT3a/b, MRF (−) or middle-upper tumor cT3a/b, cN1-2 (non-extranodal implantation), EMVI (−), while emphasizing the importance of a high-quality TME to minimize the risk of recurrence. The classical TME proposed by Heald is the “golden standard” of rectal cancer surgery and it can guarantee the surgical radicality for these patients.

**Figure 6.**
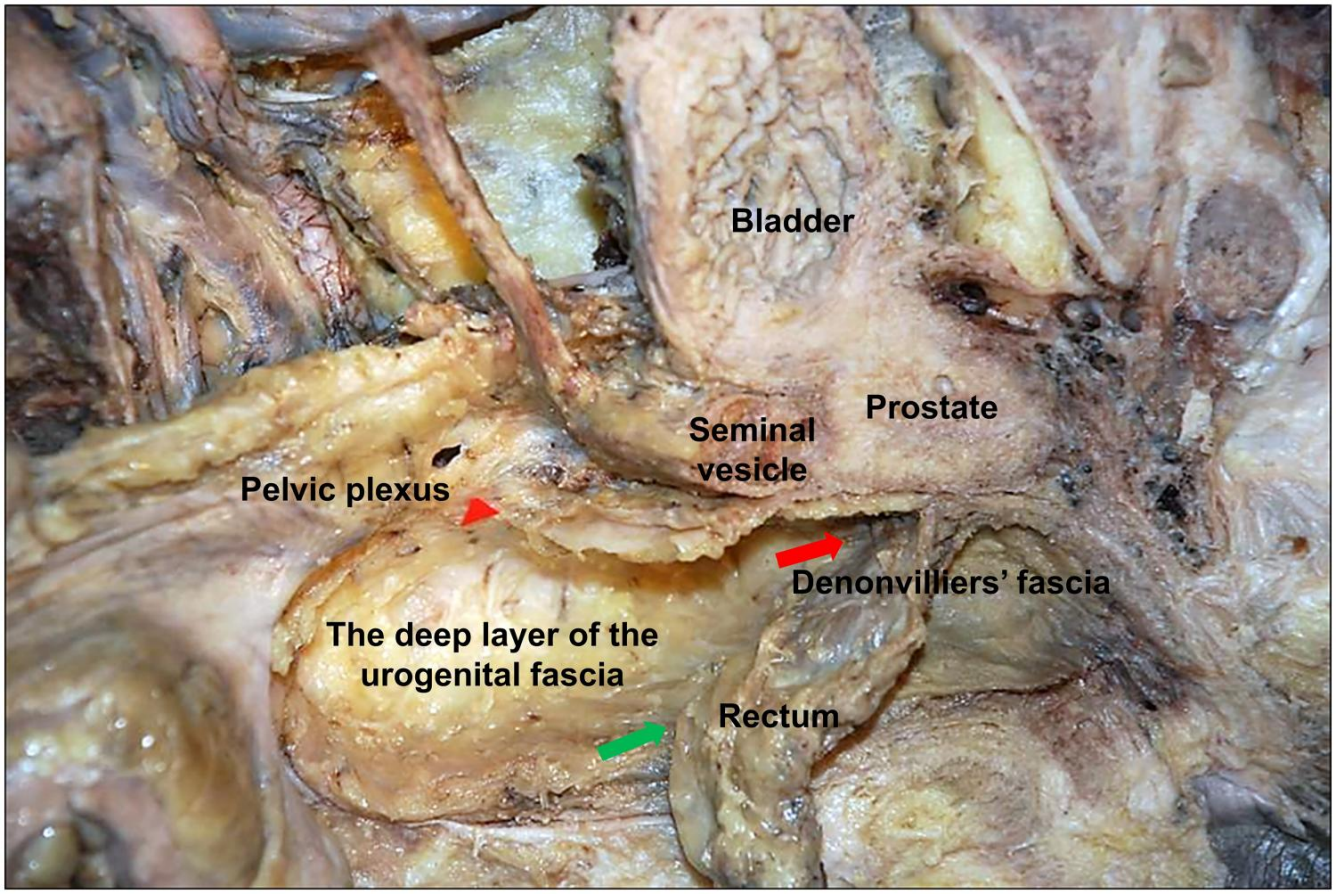
The surgical plane of TME with urogenital fascia preservation (green arrow) is located in the same space as the surgical plane of TME with Denonvilliers’ fascia preservation (red arrow)

The difference between surgery types A and B reflects a different understanding of the anatomy of the mesorectum. Type B surgery, also known as the TME surgery proposed by Heald, is based on the premise that the fascia propria of the rectum is a component of the visceral fascia (Figure 5b) [26] and that the mesorectum is surrounded by the posterolateral visceral fascia and the anterior Denonvilliers’ fascia (Figure 6). Therefore, the plane between the visceral and parietal fascia constitutes the surgical “holy plane” [1]. In contrast, type A surgery is based on the theory that the fascia propria of the rectum and the visceral fascia (urogenital fascia) are two distinct layers of fasciae [13, 19, 20, 27] and that the mesorectum is surrounded by the fascia propria of the rectum. Thus the plane surgical plane is situated between the fascia propria of the rectum and the visceral fascia. According to Heald, the visceral fascia exists as the mesorectal fascia and therefore classical TME is required to remove the visceral fascia [4]. However, in current clinical practice, almost all laparoscopic and robotic TMEs are actually type A surgeries due to the fact that the surgery preserves the urogenital fascia, which contains the HN, and the urogenital fascia and the visceral fascia have been demonstrated as the same fascia [13, 20, 27]. Consequently, this type of surgery is referred to as TME with urogenital fascia preservation [13].

The difference of the resection plane anterior to the rectum between type A and type B procedures can clarify the controversy of whether Denonvilliers’ fascia should be removed or not in clinical practice. From an anatomical standpoint, the surgical planes of TME with urogenital fascia preservation and TME with Denonvilliers’ fascia preservation are actually located in the same space (the medial space). The difference in operation names stems from the fact that the two procedures are defined from the posterolateral and anterior surgical planes (Figure 6). Both of them are actually classified as type A surgeries. Therefore, there is no need to question the clinical application of TME with Denonvilliers’ fascia preservation as long as the patient meets the indications for type A surgery.

Type B surgery can be divided into two subtypes, depending on whether or not the pelvic autonomic nerve is preserved. On the topic of function-preserving surgery, it is crucial to clarify the relationship between the urogenital fascia and the distribution of the pelvic autonomic nerves. The HN, the IHP, the VNB, and the UNB of the IHP are distributed along the deep layer of the urogenital fascia (Figure 5b), which is referred to as a “nerve plane” [28] or “nerve plate” [29] by Japanese scholars. Although there are many descriptions of nerve-sparing surgery for both rectal and cervical cancer, they can ultimately be summarized in two techniques: one is to preserve the deep layer of the urogenital fascia containing the nerves; the other is to dissect the HN, the IHP, the pelvic plexus, PSNs, and the visceral branches of the IHP from the deep layer of the urogenital fascia. The former is a common approach in laparoscopic surgery for both rectal cancer [30] and cervical cancer [29, 31]. During the whole operation process, laparoscopic surgery is performed along the space between the fasciae and there is no procedure to dissect the nerves. In contrast, the latter is the traditional approach to function-preserving surgery, as is the case with Enker’s function-preserving TME [32] and Fujii’s function-preserving hysterectomy [33]. However, the possibility of preserving the pelvic autonomic nerves is more dependent on the type of pelvic plexus rather than the radicality of the surgery. In 2011, we first proposed two types of pelvic plexus—aggregation shape and diffusion shape [34]. Traditional anatomy describes the type of aggregation shape as a dense quadrilateral structure. Because there is areolar tissue between the fascia and the pelvic plexus, it is possible to dissect and retract the nerve before excision of the deep layer of the urogenital fascia in order to complete a type B1 surgery with pelvic autonomic nerves preservation (Figure 5b). In contrast, the type of diffusion shape appears as a lamellar fibroneural network without a fixed form and is in close contact with the fascia, making it difficult to separate the nerve and fascia (Figure 5b). Even with Fujii’s precise function-preserving surgical approach [28], it is difficult to dissect and retract the nerve. Thus it is often necessary to resect the deep layer of the urogenital fascia containing the pelvic autonomic nerve to complete a type B2 surgery.

### Type C: extended TME

Two subtypes are defined: type C1 with pelvic autonomic nerve preservation and type C2 without pelvic autonomic nerve preservation.

TME and lateral lymph node dissection (LLND) are included in extended TME, which has been modified to the extent of resection outside the mesorectal plane [35]. The medial space corresponds to the resection area of the TME, while the middle and lateral spaces constitute the surgical field for LLND (Figure 5c). Thus type C is actually the surgery of three-space dissection for rectal cancer proposed by Takahashi in 2000 [18]. The concept of LLND is not applicable to gynecologic surgery, as pelvic lymph node dissection has already been incorporated into the surgery classification system. For example, LLND is an integral part of type C surgery in the Q–M classification [5]. However, LLND and TME for rectal cancer are considered to be two distinct procedures. In this paper, we also incorporated LLND into the new classification system. Because the indication for LLND in rectal cancer remains controversial, more research is needed to determine which patients will benefit from type C surgery.

Expanded TME can be further classified into two subtypes based on nerve preservation. From an anatomical point of view, “fascia-oriented” LLND [15], “fascial space priority” LLND [16], and “two spaces” LLND [17] are all type C1 procedures because they dissect the urogenital fascia to build the fascial plane, which protects the “nerve plane.” Taking the “two spaces” LLND as an example, this procedure achieves the purpose of preserving the function by preserving the urogenital fascia. We have proposed a fascia-to-space surgical approach to perform LLND. In particular, we first dissect the deep layer of the urogenital fascia, the superficial layer of the urogenital fascia, and the parietal fascia sequentially to establish the fascial planes to develop fascial spaces, and this process enables us to remove #293, #273, and #280. Then, we perform space dissection to remove #263 and #270 in the middle space and #283 and #260 in the lateral space. This technique simplifies the surgical procedure and preserves the deep layer of the urogenital fascia to accomplish nerve-sparing surgery (Figure 5c). Type C2 surgery is indicated for patients with tumors or metastatic lymph nodes invading the urogenital fascia. If preservation of the urogenital fascia would compromise the radicality, we have to remove it with autonomic nerves (Figure 5c).

### Type D: laterally extended resection

Type D is divided into two subtypes according to the extent of surgical clearance: type D1 is lateral pelvic extended radical surgery and type D2 is lateral pelvic exenteration.

In contrast to types A–C, type D is considered ultra-radical surgery and is specifically designed for locally advanced or recurrent rectal cancer. The procedure involves pelvic exenteration and the surgical value of this technique requires reevaluation. Pelvic exenteration is not only the only possible radical treatment, but also an important means to relieve pain, bleeding, and other symptoms for patients with advanced cancer. The latest results of PelvEx Collaborative indicate that the 3-year survival rate for patients with locally advanced rectal cancer can reach 56.4% if R0 resection can be achieved by pelvic exenteration [36]. Therefore, in order to achieve R0 resection, pelvic exenteration now tends towards high sacral resection or even sciatic and femoral nerve resection [37, 38].

To remove all adipose and lymphoid tissue from the lateral pelvis, type D1 requires the removal of the internal iliac vessels, including all visceral and parietal branches (Figure 5d). This type is the surgery of choice for the central-type tumor in Leeds classification [39], where the tumor is confined to the pelvic organs and does not invade the pelvic wall. Type D1 surgery is equivalent to laterally extended parametrectomy in gynecology [40].

Type D2 surgery entails the resection of adjacent fasciae, muscles, nerves, and even the sacrum of the pelvic sidewall, in addition to the procedures performed in type D1 surgery (Figure 5d). This type of radicality is the surgery of choice for patients with sacral-type, sidewall-type, or composite-type tumors in Leeds classification [39], where the tumor invades the sacrum or invades the area of the pelvic sidewall, including the greater sciatic foramen and sciatic nerve through to the piriformis and the gluteal region. In gynecology, type D2 surgery corresponds to extended lateral pelvic sidewall excision [41] and laterally extended endopelvic resection [42].

## Conclusions

The new classification presented in this paper is proposed based on the theory of “four fasciae and three spaces.” However, the main limitation of this new classification is that it depends on a clearly described anatomical and surgical concept, and requires further investigation and verification through randomized control studies. We are currently conducting a multicenter clinical study and strive to publish the preliminary results as soon as possible. The concept of membrane anatomy to determine the extent of surgical resection through fascial space proposed in this paper is consistent with Heald’s theory of the “holy plane,” which can be applied to both rectal and cervical cancer surgery. In-depth study of this idea will aid in the development of a unified and standardized classification of radical surgery for pelvic tumors.

## Author’s Contributions

J.W., H.L., A.L., and M.L. drafted the manuscript. J.W., H.L., H.J., Y.P., and X.C. reviewed and edited the manuscript. L.Y. and M.L. supervised this work. All authors read and approved the final version of this manuscript.
